# Focused Ultrasound-Induced Blood-Brain Barrier Opening: Association with Mechanical Index and Cavitation Index Analyzed by Dynamic Contrast-Enhanced Magnetic-Resonance Imaging

**DOI:** 10.1038/srep33264

**Published:** 2016-09-15

**Authors:** Po-Chun Chu, Wen-Yen Chai, Chih-Hung Tsai, Shih-Tsung Kang, Chih-Kuang Yeh, Hao-Li Liu

**Affiliations:** 1 Department of Electrical Engineering, Chang-Gung University, Taoyuan, 333, Taiwan; 2 Department of Diagnostic Radiology and Intervention, Chang-Gung Memorial Hospital, Taoyuan, 333, Taiwan; 3 Department of Biomedical Engineering and Environmental Sciences, National Tsing Hua University, Hsinchu 300, Taiwan; 4 Department of Neurosurgery, Chang Gung Memorial Hospital, Taoyuan, 333, Taiwan; 5 Medical Imaging Research Center, Institute for Radiological Research, Chang Gung University and Chang Gung Memorial Hospital, Taoyuan, Taiwan

## Abstract

Focused ultrasound (FUS) with microbubbles can temporally open the blood-brain barrier (BBB), and the cavitation activities of microbubbles play a key role in the BBB-opening process. Previous attempts used contrast-enhanced magnetic resonance imaging (CE-MRI) to correlate the mechanical index (MI) with the scale of BBB-opening, but MI only partially gauged acoustic activities, and CE-MRI did not fully explore correlations of pharmacodynamic/pharmacokinetic behaviors. Recently, the cavitation index (CI) has been derived to serve as an indicator of microbubble-ultrasound stable cavitation, and may also serve as a valid indicator to gauge the level of FUS-induced BBB opening. This study investigates the feasibility of gauging FUS-induced BBB opened level via the two indexes, MI and CI, through dynamic contrast-enhanced (DCE)-MRI analysis as well as passive cavitation detection (PCD) analysis. Pharmacodynamic/pharmacokinetic parameters derived from DCE-MRI were characterized to identify the scale of FUS-induced BBB opening. Our results demonstrated that DCE-MRI can successfully access pharmacodynamic/pharmacokinetic BBB-opened behavior, and was highly correlated both with MI and CI, implying the feasibility in using these two indices to gauge the scale of FUS-induced BBB opening. The proposed finding may facilitate the design toward using focused ultrasound as a safe and reliable noninvasive CNS drug delivery.

The blood–brain barrier (BBB) is a major limitation for the treatment of central nervous system (CNS) diseases because it blocks molecules with molecular weights exceeding 400 Da^1^,^2^,^3^. Unfortunately, most potent therapeutic agents exceed this size and are thus blocked by the BBB. Low-intensity burst-type focused ultrasound (FUS) combined with microbubbles administration has recently been shown to open the BBB in a non-invasive, localized, and transient manner^4^,^5^, raising new possibilities for delivering therapeutic agents directly into the brain.

Before these developments can be translated into clinical applications, indexes must be developed to gauge the likelihood of the FUS-induced BBB opening, so that CNS therapeutic molecule delivery can be well gauged and estimated. A number of preclinical studies have discussed FUS-induced BBB opening with different FUS parameters including exposure frequency, acoustic pressure, burst length, pulse-repetition frequency, duration and microbubble dose^6^,^7^,^8^,^9^,^10^. In addition, the mechanical index (MI), defined as peak negative acoustic pressure over the square root of the frequency (i.e., MI = P/√f, P in MPa, f in MHz), has potential to serve as an index to gauge ultrasound-induced mechanical bio-effects^11^. Previously, McDannold *et al*. found a high correlation between FUS-induced BBB opening with MI by employing signal intensity (SI) change of contrast-enhanced magnetic resonance imaging (CE-MRI). They were able to identify thresholds to indicate the occurrence of FUS-induced BBB opening^8^. Subsequent studies described the correlation of MI level with the occurrence erythrocyte extravasations, which is the primary adverse effect in this BBB-opening process^12^,^13^,^14^. While recent studies have reported the association of MI with FUS-induced BBB opening, the level of MI is usually considered to reflect the scale of inertial cavitation^11^.

It has long been understood that inertial and stable microbubble-present acoustic cavitation can be characterized from distinct backscattered acoustic emissions. Inertial cavitation can be characterized by wideband emissions, and refers to microbubble collapses and disruptions; stable cavitation can be characterized by subharmonic/ultraharmonic emissions, and refers to the stable contraction/expansion of microbubbles^15^,^16^. Passive cavitation dose (PCD) analysis is used to detect and characterize backscattered acoustic emissions to reflect microbubble activities that accompany FUS-induced BBB opening, and a growing number of recent studies suggest that the occurrence of FUS-induced BBB opening not only relates to inertial cavitation but more likely is caused by stable cavitation^14^,^17^,^18^,^19^.

To estimate the scale of stable cavitation, recently Bader *et al*. derived the cavitation index (CI), defined as peak negative acoustic pressure (in MPa) over frequency (in MHz); i.e., CI = P/f), to gauge the likelihood of subharmonic emissions due to microbubble-presented stable cavitation activity^15^. Unfortunately, so far no reports show the feasibility in using CI as a gauge to measure the scale of FUS-induced BBB opening, nor a clear evaluation in comparing the effectiveness between using MI and CI to gauge this effect.

This study assesses the use of MI and CI as indicator of FUS-induced BBB opening. Unlike CE-MRI, dynamic-contrast-enhanced (DCE) MRI provides pharmacodynamic and pharmacokinetic analysis capability (when using Gd-DTPA (molecular weight of 961 Da) as a surrogate of the delivered molecule) and can better describe the FUS-induced BBB-opening process. To investigate this, we monitored the change of pharmacodynamic (PD)/pharmacokinetic (PK) parameters measured from DCE-MRI *in vivo*. Various FUS parameters obtained from different combinations of frequency and pressure exposure were designed to correspond to changing MI/CI levels, and association between indexes with BBB-opening levels were evaluated. Kinetic measurements were also performed at multiple time points after FUS exposure to evaluate whether the correlations could hold for the entire BBB-opening/closing period. PCD analysis was also used to characterize the types of acoustic emissions to support our conclusions.

## Results

### DCE-MRI analysis to access FUS-induced BBB opening

**Figure 1:**
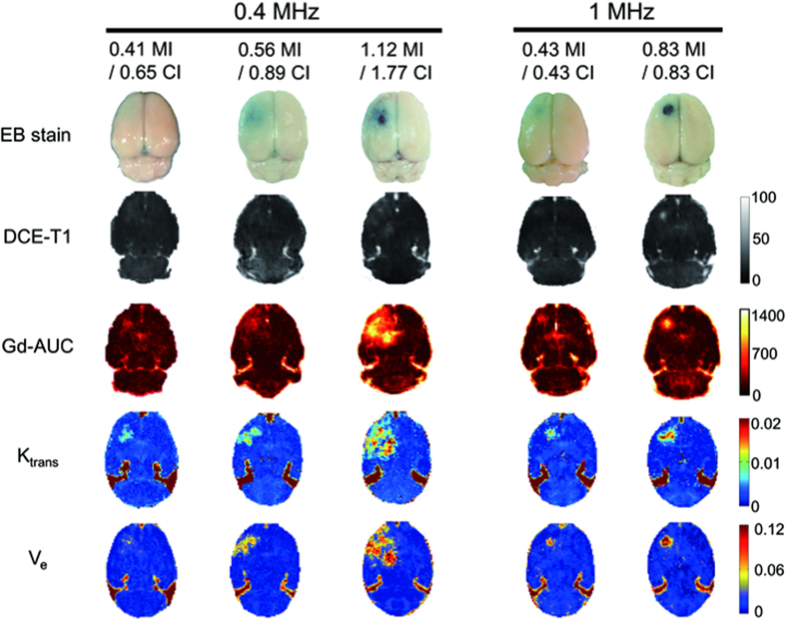
Representative gross views of EB-stained brains and post-processed DCE-MRI parameters including the signal-intensity (SI) maps, Gd-based area-under-curve (Gd-AUC) maps, K_trans_ maps, and V_e_ maps at various MI/CI exposure levels. The scale of BBB-opening increases with MI/CI for both the 0.4-MHz FUS group and the 1-MHz FUS group. The mild BBB-opening caused by low MI/CI with 1-MHz FUS was similar to the BBB-opening of low MI/CI 0.4-MHz FUS. The higher MI/CI 0.4-MHz FUS and higher MI/CI 1-MHz FUS induced aggressive BBB-opening accompanied by erythrocyte extravasations. The FUS dimension is larger in 0.4-MHz than in l-MHz, therefore 0.4-MHz exposure contributed to a larger BBB-opening dimension.

To examine PD/PK parameters under various FUS settings, we tested FUS with different combinations of exposure frequency (either 0.4 or 1 MHz) and pressure (0.25–0.83 MPa) to produce exposure level in the range of 0.41–1.12 when gauged by MI, and 0.43–1.77 when gauged by CI. Animals received a single FUS exposure (10 ms bursts length, 1 Hz pulse pulse-repetition frequency, 90 s exposure duration, 0.2 mL/kg SonoVue^®^) for evaluation (animal experimental setup is shown in Supplementary Fig. S1A). Following the DCE-MRI, the animals were sacrificed and their brains were stained with Evans blue dye. The selection of MI and CI levels cover a wide spectrum of known biological and pathological effects that range from intact BBB-opening to erythrocyte extravasations (see Supplementary Table S1). Figure 1 summarizes the comparison of the EB-stained brains with post-processed DCE-MRI parameters, including the signal-intensity (SI) maps, Gd-based area-under-curve (Gd-AUC) maps, K_trans_ maps, and V_e_ maps at various MI/CI exposure levels. The EB-stained brains confirmed the opening of the targeted BBB and correlated with the locations of all four maps. The scale of BBB-opening was found to be dependent on MI/CI level changes. A low MI/CI value (0.41-MI/0.65-CI and 0.56-MI/0.89-CI at 0.4-MHz exposure; 0.43-MI/0.43-CI at 1-MHz exposure) induced a mild BBB-opening with light EB-staining and results in similar level changes of DCE-MRI maps, whereas high MI/CI values (1.12-MI/1.77-CI of 0.4-MHz FUS; 0.83-MI/0.83 CI of 1-MHz FUS) induced more aggressive BBB-opening with increased EB leakage, accompanied by erythrocyte extravasations, along with higher changes of DCE-MRI maps. In addition, the change of exposure frequency resulted in various BBB-opening dimensions due to the frequency-dependent focal dimension change (FUS dimension is larger at 0.4-MHz than at l-MHz, therefore 0.4-MHz exposure contributed to a larger BBB-opening), but was found to be independent of the scale of the FUS-induced BBB opening.

### DCE-MRI to characterize pharmacodynamic (PD) behaviors of FUS-induced BBB opening

**Figure 2:**
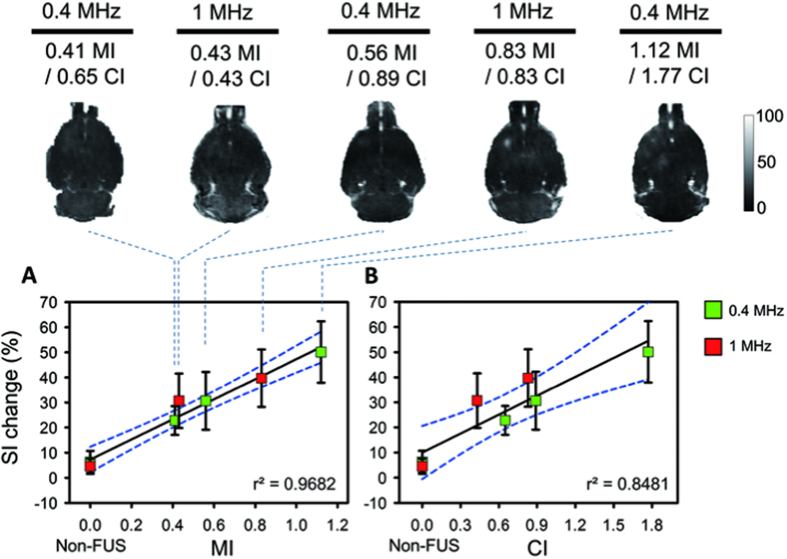
Gd-DTPA enhanced T1-weighted maps and correlations of MI/CI with SI change within 10 min. SI change was increased as a function of MI/CI change. (**A**) The correlation between MIs and SI changes. The non-FUS side serves as 0 MI. The SI increase was monotonically increased as a function of MI change regardless of exposure frequency. (**B**) The correlation between CIs and SI changes. The non-FUS side serves as 0 CI. The correlation of CI and SI change decreased but was still sufficiently high.

SI change obtained from DCE-MRI analysis after Gd-DTPA administration is illustrated in Fig. 2 as a reference for the subsequent PD analysis, and the correlation of SI change between MI and CI is separately shown in Fig. 2A,B (detailed estimations are summarized in Supplementary Table S2). When gauged by MI, the SI increases monotonically as a function of MI change regardless of exposure frequency (SI change value increases from 22.806% to 50.134%), and a high correlation between SI and MI can be observed (r^2^ = 0.9682). In contrast, the correlation of CI with SI change falls slightly but is still sufficiently high (r^2^ = 0.8481) since the SI change in 1-MHz exposure is higher than for the 0.4-MHz exposure given similar CI exposure levels. Correlation of MI versus SI seems relatively outperformed than correlation of CI versus SI but without significance (Z = 1.15, *p* = 0.25, two-tailed via Fisher’s r to z transformation; see Supplementary Table S3).

**Figure 3:**
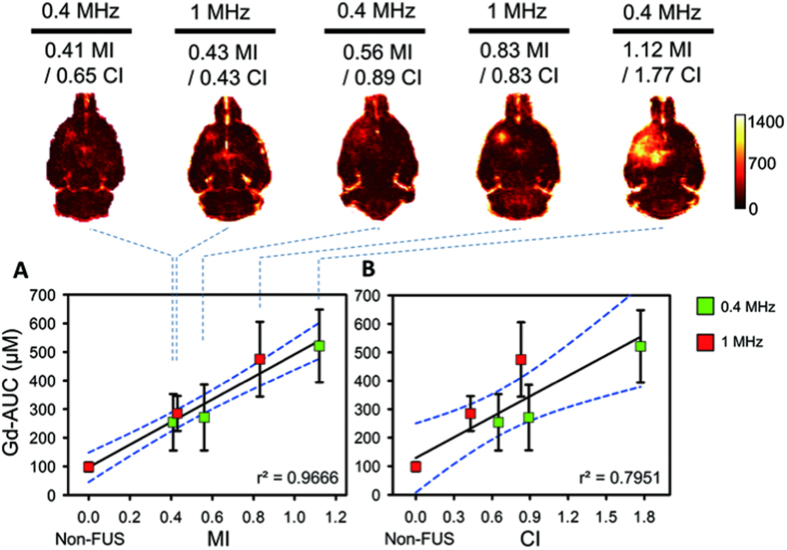
Post-processed Gd-AUC maps and correlations of MI/CI with Gd-AUC change within 60 min. Gd-AUC was increased as a function of MI/CI change. (**A**) Correlation between MIs and Gd-AUCs. The non-FUS side serves as 0 MI. The Gd-AUC was monotonically increased as a function of MI change regardless of exposure frequency. (**B**) Correlation between CIs and Gd-AUCs. The non-FUS side serves as 0 CI. The correlation of CI with Gd-AUC was slightly degraded but still sufficiently high.

The correlations between FUS-induced BBB-opened level with MI/CI were then accessed by DCE-MRI PD analysis. In DCE-MRI PD analysis, the Gd-AUC maps were obtained to access BBB-opening by analyzing Gd-DTPA enhanced T1-weighted images following 60 min of Gd-DTPA administration (the sequential time line of experiments is shown in Supplementary Fig. S1B). Figures 2 and 3 can be used to comparing the SI change and the analysis results of post-processing Gd-AUC to characterize the PD behavior of FUS-induced BBB-opening. Compared with the SI change, Gd-AUC was also monotonically increased by exposure level when gauged by MI, (with Gd-AUC value increasing from 253.962 μM·min to 521.063 μM·min) and resulted in a high degree of correlation with MI level (r^2^ = 0.9666; Fig. 3A). In contrast, when gauged by CI, a smaller but still sufficiently high correlation can be found between Gd-AUC and CI (r^2^ = 0.7951; Fig. 3B). The accumulated Gd obtained in Gd-AUC was relatively higher following 1-MHz exposure than 0.4-MPa exposure at similar CI exposure levels. For example, 0.83-CI at 1 MHz contributes to 474.83 μM·min of Gd accumulation whereas 0.89-CI at 0.4 MHz contributes to only 270.776 μM·min. This contributes to a slightly reduced but still sufficiently high correlation between Gd-AUC and CI (r^2^ = 0.7951). Correlation of MI against Gd-AUC seems relatively outperformed than correlation of CI against Gd-AUC but without significance (Z = 1.35, *p* = 0.177, two-tailed via Fisher’s r to z transformation; see Supplementary Table S3). Therefore, both MI and CI could be considered as valid indexes to assess the PD behaviors of FUS-induced BBB-opening.

### DCE-MRI to characterize pharmacokinetic (PK) behaviors of FUS-induced BBB opening

**Figure 4:**
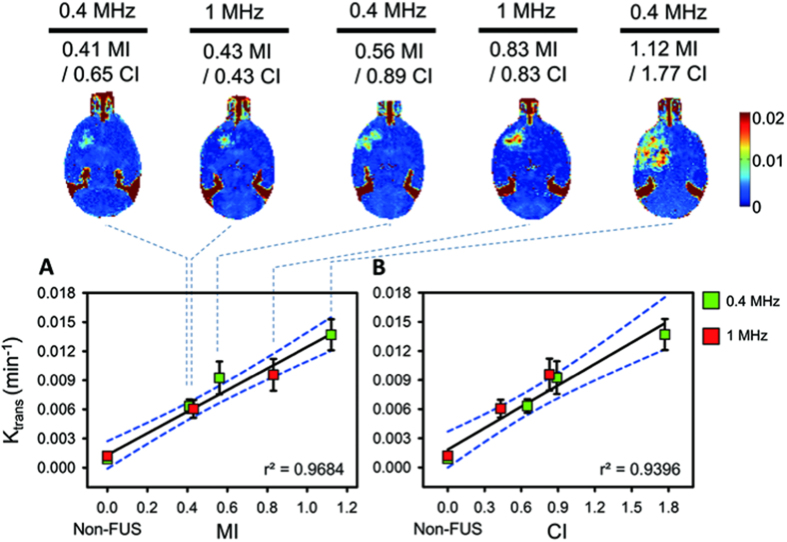
Post-processed K_trans_ maps and correlations of MI/CI with K_trans_ change within 10 mins. K_trans_ was increased as a function of MI/CI change. (**A**) Correlation between MIs and K_trans_s. The non-FUS side serves as 0 MI. The K_trans_ was monotonically increased as a function of MI change regardless of exposure frequency. (**B**) Correlation between CIs and K_trans_s. The non-FUS side serves as 0 CI. The correlation of CI with K_trans_ was still sufficiently high.

The correlations between degree of FUS-induced BBB opening with MI/CI were then determined by DCE-MRI PK analysis. DCE-MRI PK parameters including K_trans_ and V_e_ were obtained by analyzing Gd-DTPA enhanced T1-weighted images following 10 min of Gd-DTPA administration. The PK parameter, K_trans_, was evaluated first. Compared to the non-FUS side brain, the value clearly increased from a low to high FUS level (K_trans_ level increased from 0.0061 to 0.0136 min^−1^ with MI exposure level from 0.41- to 1.12-MI) and presented a high correlation with MI (r^2^ = 0.9684; Fig. 4A). On the other hand, K_trans_ was also observed to be highly correlated with CI exposure level (r^2^ = 0.9396) and to be less dependent on exposure frequency (0.43- to 0.83-CI at 1-MHz exposure contributed to K_trans_ from 0.0061 to 0.0095 min^−1^, which was very similar with the K_trans_ level change from 0.0063 to 0.0092 min^−1^ for 0.65- to 0.89-CI FUS of 0.4-MHz exposure; see Fig. 4B). The Correlated level of MI against K_trans_ was almost equivalent with the correlation of CI against K_trans_ (Z = 0.47, *p* = 0.638, two-tailed via Fisher’s r to z transformation; see Supplementary Table S3).

**Figure 5:**
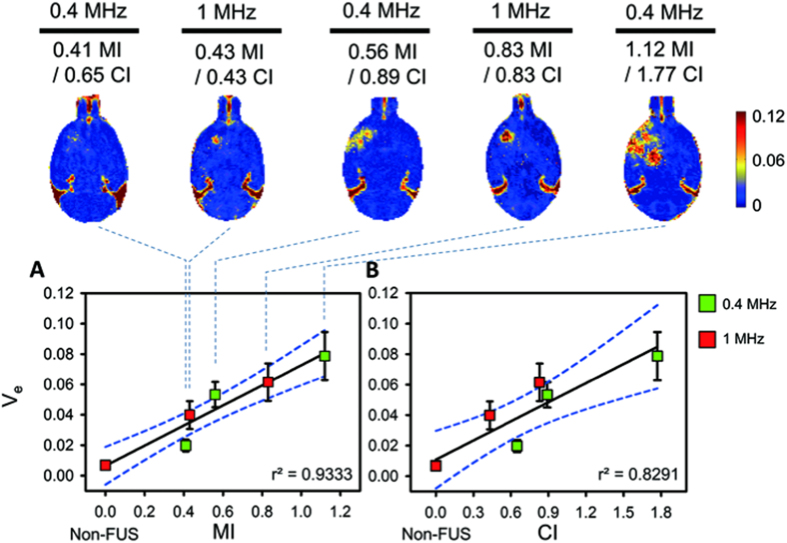
Post-processed V_e_ maps and correlations of MI/CI with V_e_ change within 10 min. V_e_ was increased as a function of MI/CI change. (**A**) Correlation between MIs and V_e_s. The non-FUS side serves as 0 MI. The V_e_ was monotonically increased as a function of MI change regardless of exposure frequency. (**B**) Correlation between CIs and V_e_s. The non-FUS side serves as 0 CI. The correlation of CI with V_e_ was slightly lower but still sufficiently high.

The correlations of the second PK parameter, V_e_, under MI/CI were then evaluated. The correlations of V_e_ with MI was still high compared to K_trans_ (r^2^ = 0.9333; V_e_ value from 0.0285 to 0.0787 when MI exposure level varied from 0.41- to 1.12-MI; see Fig. 5A). On the other hand, the correlation between V_e_ and CI was slightly less than that with MI (r^2^ = 0.8291; see Fig. 5B) due to the more diverse V_e_ distribution for similar CI exposure levels at two exposure frequencies (For example, 0.43- to 0.83-CI of 1-MHz exposure contributed to V_e_ changing from 0.0398 to 0.0616, whereas 0.65- to 0.89-CI of 0.4-MHz exposure contributed to a slightly drop in V_e_ values from 0.0285 and 0.0533). Correlation of MI against V_e_ seems relatively outperformed than correlation of CI against V_e_ but without significance (Z = 0.71, *p* = 0.211, two-tailed via Fisher’s r to z transformation; see Supplementary Table S3). In general, the results of the four imaging parameters under the five exposure conductions are summarized in Supplementary Table S2. It shows that the four DCE-MRI parameters can well correspond to the exposure level change and can well correlate with MI and CI.

**Figure 6:**
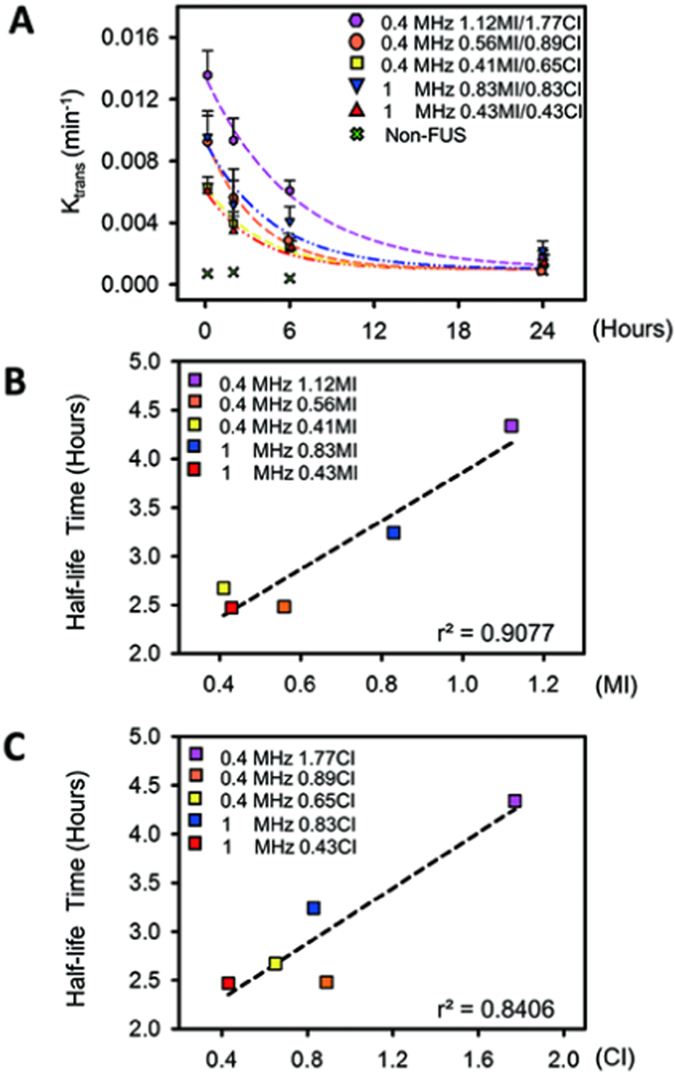
Time-dependent changes of K_trans_ obtained from regions of FUS-induced BBB opening at four time points (10 min, 2 hr, 6 hr, and 24 hr). (**A**) K_trans_ exponential decay curve for each MI/CI subgroup. The decay time was prolonged with the increased MI/CI FUS regardless of exposure frequency. (**B**) Correlation between MIs and K_trans_ half-life time from BBB-opening to BBB-closure. The half-life times were highly correlated with MIs regardless of exposure frequency. (**C**) Correlation between CIs and K_trans_ half-life time of BBB-opening to BBB-closure. The half-life times were well correlated with CIs regardless of exposure frequency.

We next assessed the correlation of MI-CI level with DCE-MRI kinetic parameters for the entire BBB-opening/closure process to test the correlation with BBB recovery. To investigate the correlation for the entire BBB-opening/closured period, PK analysis was repeated at four time points after initial FUS exposure (10 min, 2 hr, 6 hr, and 24 hr). The sequential time line of the experiments is shown in Supplementary Fig. S1B. Since the above analysis revealed that K_trans_ obtained sufficiently high correlations for either MI or CI, it was selected from among the four PD/PK parameters to evaluate correlations of BBB-opening transients with MI/CI (dynamic changes of K_trans_ at various FUS parameters are shown in Fig. 6A). K_trans_ values were generally shown to fall over time, indicating that the FUS-induced BBB opening was reversed and closed gradually.

The hourly half-lives of the PK parameters were estimated to represent the rate of BBB-closure and are summarized in Supplementary Table S2. The BBB-opening half-life was found to be both CI and MI dependent, with greater exposure (gauged either by MI or CI) contributing to a longer half-life, thus extending the leaky status of the FUS-exposed capillaries. A similar K_trans_ half-life could be identified in the intermediate FUS exposure (2.47–2.67 hrs) that induced intact BBB-opening in one group, while excessive FUS exposure induced extensive extravasations with the identified longer half-life time (3.24–4.34 hrs) in another. The correlations of estimated of K_trans_ half-life times are shown in Fig. 6B,C, and are observed to both correlate well when either gauged by MI (r^2^ = 0.9077) or by CI (r^2^ = 0.8406) (Z = 0.29, *p* = 0.7718, two-tailed via Fisher’s r to z transformation).

### Passive cavitation detection (PCD) to characterize FUS-induced BBB opening

**Figure 7:**
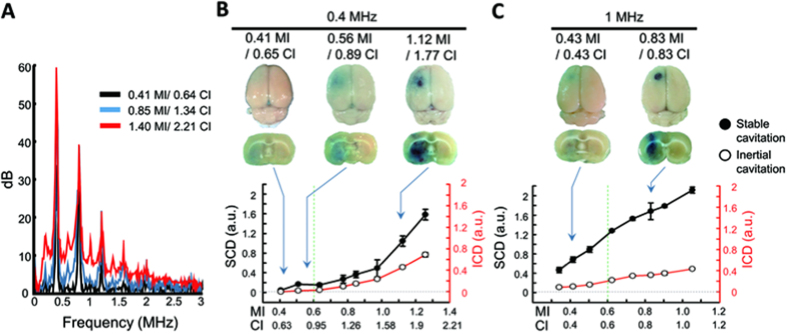
PCD analysis from inertial cavitation and stable cavitation during various FUS levels. (**A**) Typical spectrum change of backscattered signals with increased MI/CI. The subharmonic/ultraharmonic emissions and wideband emissions increased with MI/CI. (**B**) SCD and ICD of 0.4-MHz burst-FUS. The SCD occurred before the ICD and both doses increased with FUS exposure level. (**C**) SCD and ICD of 1-MHz burst-FUS. SCD and ICD both increased with the FUS exposure level, indicating that stable and inertial cavitation could be enhanced by and dependent on FUS level.

Inertial cavitation and stable cavitation can be characterized by acoustic emissions. To identify their association with BBB-opening and with MI and CI, passive cavitation detection (PCD) analysis was conducted to determine the role of stable cavitation (quantified by stable cavitation dose, SCD) and inertial cavitation (quantified by inertial cavitation dose, ICD). The FUS parameters in these *ex vitro* experiments were selected to be identical with the *in vivo* experiments (considering transcranial pressure loss) so that the PCD analysis and the DCE-MRI PK/PD analysis could be mutually correlated. Figure 7A shows the typical detected spectrums of backscattered signals. The components of either wideband or subharmonic/ultraharmonic emissions increased with the FUS exposure level.

Figure 7B,C respectively show the tendencies of PCD signals (SCD/ICD) and MI/CI when quantifying cavitation activities to SCD and ICD as function of MI/CI. Generally, SCD and ICD both increased with the FUS exposure level, indicating the occurrence of stable and inertial cavitation is enhanced by and dependent on FUS level. However, in the mild FUS exposure range, the increase of BBB-opening was more likely dependent on the SCD increase. For example, for 0.4-MHz exposure, the FUS level increased from 0.65- to 0.89-CI, the BBB-opening level (identified by the increased EB staining) with SCD was apparently increased from 0.044 to 0.167 a.u. (an increase of 273%), whereas the ICD tended to remain relatively stable (from 0 to 0.015). For exposure levels exceeding exceed 0.6-MI, both the 1- and 0.4-MHz FUS exposure induced noticeable erythrocyte extravasations, accompanied by FUS-induced BBB-opening (with histology results shown in Supplementary Fig. S2), and ICD changed dramatically (272% and 850.7% change in 1- and 0.4-MHz exposure, respectively).

### Association of FUS-induced BBB opening when gauged by MI and CI

**Figure 8:**
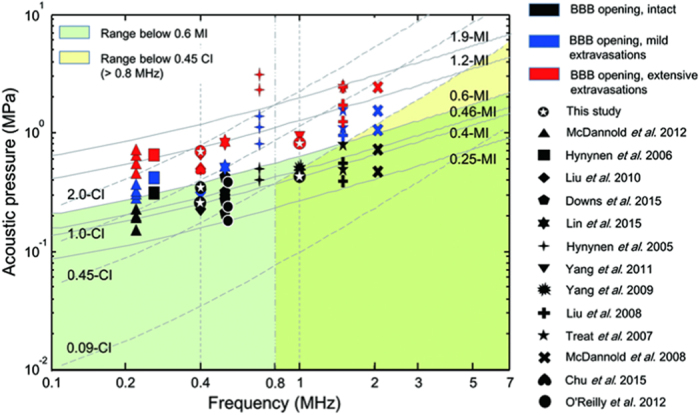
FUS-induced BBB opening of the previously employed exposure levels on the iso-contour MI/CI lines and denoted as three different levels: intact (marked in black), mild erythrocyte extravasations (marked in blue), and severe erythrocyte extravasations/brain damages (marked in red). (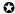 This study, ▴ McDannold *et al*.^27^, ◾ Hynynen *et al*.^20^, 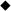 Liu *et al*.^23^, 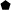 Downs *et al*.^28^, 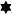 Lin *et al*.^26^, 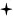 Hynynen *et al*.^6^, ▾ Yang *et al*.^25^, 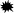 Yang *et al*.^24^, 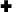 Liu *et al*.^22^, 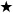 Treat *et al*.^21^, 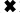 McDannold *et al*.^8^, 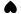 Chu *et al*.^12^). Specifically, FUS exposure levels within the range below 0.6-MI (green area) and below 0.45-CI (above 0.8 MHz, yellow area) seemed to induce intact BBB-opening without significant erythrocyte extravasations or brain damage. Note: exposure level of 1.9-MI reaches the maximum limit from diagnostic ultrasound purposed regulation^47^.

To assess whether the FUS-induced BBB-opening can be accurately gauged by either MI or CI, Fig. 8 overlays the applied exposure levels on the previously used iso-contour MI/CI lines^15^, using three different levels of BBB-opening: intact (marked in black), mild erythrocyte extravasations (marked in blue), and severe erythrocyte extravasations/brain damages (marked in red). Meanwhile, to consider additional correlations, Fig. 8 summarizes the results of recent studies using similar FUS exposures (i.e., burst length = 10 ms, PRF = 1 Hz)^6^,^8^,^12^,^19^,^20^,^21^,^22^,^23^,^24^,^25^,^26^,^27^,^28^. With the MI iso-curve ranging from 0.25 to 1.9 (solid lines), 0.25-MI was the lowest previously-reported exposure level for BBB opening^8^,^22^,^28^, and 0.25- to 0.6-MI is the range in which sufficiently stable cavitation activity is induced. This result is consistent with previously reports of 0.46-MI having an 80% probability threshold of BBB-opening^8^, below the point at which significant inertial cavitation could be detected (0.6-MI)^11^. Specifically, FUS exposure below 0.6-MI (zone marked in light green) could induce intact BBB-opening without significant erythrocyte extravasations or brain damage; for exposures exceeding 0.6-MI, consistent extravasations were associated with the FUS-induced BBB opening and it seems the increased extravasation scales was associated with the increase of MI level. On the other hand, when overlaying these reported BBB-opening levels with CI iso-contours (dashed lines; ranging from 0.09- to 2-CI), a specific margin can also be identified (below 0.45 CI, above 0.8 MHz; zone marked in light yellow) to characterize the margin below extensive BBB opening (i.e., accompanying with extensive extravasations). MI is observed to be appropriate to gauge the level of FUS-induced BBB opening, particularly when one seeks to gauge the accompanying adverse effects such as extensive erythrocyte extravasations or brain damage (MI > 0.6). In addition, when limiting the applicable frequency range >0.8 MHz, 0.45-CI also serves as an effective indicator to gauge the MI-estimated BBB-opened likelihood.

## Discussion

Three DCE-MRI parameters (SI Change, Gd-AUC, V_e_) were found have sufficiently high correlations both with MI and CI, but was generally found to have superior correlations in MI (r^2^ = 0.93–0.96) than in CI (r^2^ = 0.79–0.84). Bader *et al*. in their theoretical derivation claimed that CI cannot accurately quantify stable cavitation below 0.8 MHz due to the theoretical limit to allow free harmonic microbubble expansion/contraction^15^. In addition, it should be aware that, when exceeding 0.45 CI, inertial cavitation can be extensively involved and solely considering CI (primarily for stable cavitation indication) as a single gauging index may not be proper. This is supported by when singly considering 1-MHz exposure condition the correlation in 0.45 CI was increased (r^2^ > 0.94; see Supplementary Table S3). These limitations may explain why MI outperformed the gauging of BBB-opening level in our DCE-MRI analysis. In general, both MI and CI could generally be considered as valid gauging indexes when accessing the pharmacokinetic/pharmacodynamic behaviors of FUS-induced BBB opening through DCE-MRI. Gauging FUS-induced BBB opening via CI should be also durable as MI, but may need to consider its applicable range together.

This study employed DCE-MRI tool to investigate the correlation between the FUS-induced BBB-opened level with MI and CI, and Gd-DTPA leakage was served as a surrogate in testing the BBB-opened scale. The advantage is that Gd-DTPA intrinsically blocked by BBB and its penetration (after BBB being opened) directly changes the T1-weighted MRI signal level. We have previously demonstrated that DCE-MRI technique is capable to semi-quantitate Gd-DTPA^29^,^30^,^31^. Here, we also quantitated Evans blue concentration to reconfirm the correlation of the EB concentration with the MI/CI (see Supplementary Fig. S3). We reconfirmed that EB deposition level was also highly correlated with MI and CI (r^2^ = 0.9227 and 0.7634, respectively; Z = 0.86, *p* = 0.3898, two-tailed via Fisher’s r to z transformation) and consistent with our previous report^31^. Based on the above results, the proposed DCE-MRI parameters should be qualified to serve as an *in-vivo* indicator to portray the level of FUS-induced BBB opening.

CE-MRI is already recognized as useful tool for the post-op evaluation of the degree and region of FUS-induced BBB opening via signal intensity change in T1-weighted images^4^,^6^,^23^,^32^. However, it does not allow for dynamic and kinetic analysis to provide a detailed understanding of transient blood-to-brain permeability change. In contrast, dynamic CE-MRI (DCE-MRI) has been proposed to provide a comprehensive description of dynamic change of FUS-induced BBB opening by calculating the pharmacodynamic (PD) and pharmacokinetic (PK) parameters when Gd-DTPA is deposited in the BBB-opening region^30^,^31^,^33^,^34^. Previous preclinical studies have proposed the use of spin-spin relaxometry (R1) and Gd-based area-under-curve (Gd-AUC) to precisely characterize PD changes of the BBB-opening region around a tumor after FUS^30^. In addition, PK parameters such as K_trans_ (which describes the influx transfer constant between extracellular extravascular space (EES) and blood plasma) or V_e_ (which describes the EES fractional volume) can describe dynamics from BBB-opening to BBB-closure^17^,^31^. Thus it is believed that DCE-MRI analysis provides information superior to that of SI change from CE-MRI to determine the accuracy of CI or MI in gauging FUS-induced BBB opening.

From the PCD analysis shown in Fig. 7, FUS exposure parameters in the range of MI level ϵ [0.41, 0.6] showed that only SCD change was detected but no ICD changes. This implies that the inertial cavitation has not been enhanced at this FUS exposure level, and that the FUS-induced BBB opening relies purely on stable cavitation. In contrast, exposure levels exceeding this range (reaching 1.12-MI for 0.4-MHz or 0.83-MI for 1-MHz) corresponded with increases to SCD and ICD. This implies that both inertial and stable cavitation are involved in the BBB-opening process at this high exposure frequency level. In addition, the occurrence of erythrocyte extravasations at this exposure level suggests that such extravasations are due to enhanced inertial cavitation^35^. This finding supports our previous observation^36^, and those of other research group^37^,^38^.

In using the SI change obtained from Gd-enhanced MRI as a reference, we have shown that the SI change in describing the BBB-opening scale is well correlated with MI/CI (Fig. 2; MI: r^2^ = 0.9682; CI: r^2^ = 0.8481). With similar FUS exposure parameters, the results of our reported FUS-induced BBB opening scale/level corresponds well with previously reported results (see Fig. 8). When the comparisons are further expanded with more diverse FUS exposure parameters (including duration ranging from 20 to 90 s, burst length ranging from 10–50 ms and multiple FUS exposures), neither MI nor CI accurately gauged the BBB-opening level (r^2^ both less than 0.2)^6^,^8^,^20^,^21^,^22^,^23^. However, after performing a scaled transformation to unify exposure times (via the transform of: SI = 40.07 · MI × t1/t2 + 7.24 and SI = 25.15 · CI × t1/t2 + 9.99, where t1 is exposure duration applied in other studies and t2 is the exposure duration applied in this study), the obtained correlation level can be improved (gauged by MI: r^2^ = 0.5853, gauged by CI: r^2^ = 0.5853; see Supplementary Fig. S4). This supports the potential in further unifying other FUS exposure parameters and can be further investigated.

Aside from comparing SI changes in CE-MRI analysis with previous studies, we also showed that 60 min of Gd-AUC accumulation can provide accurate predictions of the accumulation of large molecules (albumin-bounded EB; 70-kDa molecule)^30^. We have previously shown that Gd-AUC reached 312 μM·min for 0.63-MI/0.99-CI FUS exposure and was identify to hold close estimations performed in this study. Further reducing the AUC estimation time would increase feasibility for future clinical practice, but may degrade its superiority in evaluating pharmacodynamic/drug-accumulated features.

For kinetic analysis, we previously reported that the K_trans_ ranged 0.0086–0.0131 min^−1^ and V_e,_ ranged 0.0431–0.0692 (with the MI ranged 0.63–1.26) at the applied FUS exposure levels^31^. These measurements are in good agreement with the predicted level performed in this study (K_trans_ ranged 0.0082–0.0166 min^−1^ and V_e_ ranged 0.0481–0.096 with the MI ranged 0.63–1.26). In addition, Park *et al*. reported that K_trans_ ranged 0.0086–0.0232 min^−1^ exposure at 0.96-MI^33^ and our predicted values (0.0118 min^−1^) also closely corresponded and matched with their measurements.

## Conclusion

This study uses DCE-MRI analysis to investigate the association of the two important indexes, MI and CI, and evaluates their effectiveness for gauging FUS-induced BBB opening. While microbubbles play a significant role in the process of FUS-induced BBB opening, the level of inertial cavitation and stable cavitation involved in the microbubble-ultrasound interaction must be evaluated to guarantee BBB-opening level and quality. MI and CI serve the major indicators in gauging inertial and stable cavitation activity respectively. It therefore would be valuable to use both MI and CI to understand the roles of two distinct cavitation sources on FUS-induced BBB-opening. We demonstrated that levels of BBB-opening PD/PK changes as well as the recovered dynamics were all correlated with MI and CI, indicating that both indexes can serve as effective indicators to gauge the BBB-opening. Using DCE-MRI to assess MI or CI is seen as an effective way to control the scale of BBB-opening, and may provide a useful approach for the development of safe, reliable, noninvasive CNS drug delivery.

## Materials and Methods

### FUS Instrumentation

The FUS instrument consists of a function generator (33120A, Agilent, Palo Alto, CA, USA), a power amplifier (150A100B, Amplifier Research, Souderton, PA, USA) and a 0.4-MHz FUS transducer (Imasonic, France; diameter = 60 mm, radius of curvature = 80 mm, and electric-to-acoustic efficiency = 70%) or a 1-MHz FUS transducer (RK-300, FUS Instruments, Toronto, Ontario, Canada; diameter = 25 mm, radius of curvature = 20 mm, and electric-to-acoustic efficiency = 73%). The setup for animal experiments is shown in Supplementary Fig. S1A. Transducers were measured in a free field filled with deionized/degassed water by a needle type hydrophone. The diameter and length of the half-maximum acoustic pressure of the FUS field were 2 and 10 mm for 0.4-MHz FUS; 1.2 and 9.8 mm for 1-MHz FUS. The transcranial pressure loss was also measured with an *ex vivo* rat skull placed between the transducer and hydrophone.

### Animal Experiments

Experiments were carried out in accordance with the approved guidelines for the Care and Use of Laboratory Animals (NIH publication no. 86–23, revised 1987). All experimental protocols were approved by the Institutional Animal Care and Use Committee (IACUC) of Chang Gung University and performed according to ARRIVE (Animal Research: Reporting *In Vivo* Experiments) guidelines for the care and use of laboratory animals. A total of 28 animals (male Sprague-Dawley rats, 250–300 g, aged 8 weeks) were randomly assigned to the experimental groups. Animals received burst-mode FUS at anterior-posterior (AP) 0 mm and midline (ML) −3.5 mm from bregma following the administration of SF6-coated microbubbles. To characterize the FUS-induced BBB opening, Evans blue dye and Gd-DTPA were administrated intravenously following FUS.

Various combinations of exposure frequency and pressure (0.32–0.88 MPa for 0.4-MHz FUS and 0.75–1.46 MPa for 1-MHz FUS) were used to characterize the scale of BBB-opening. With transcranial pressure loss in the rat skull (20% for 0.4-MHz FUS and 43% for 1-MHz FUS), 0.41–1.12 MI and 0.43–1.77 CI were tested to evaluate the association between MI/CI and BBB-opening levels. These parameters covered a spectrum of known biological and pathological effects of FUS-induced BBB opening from intact BBB-opening to aggressive BBB-opening with erythrocyte extravasations^8^,^12^,^23^,^27^,^31^,^32^,^39^.

To investigate the scale of BBB-opening for various MI/CI FUS levels, microbubbles were administered to five experimental groups with 0.4-MHz FUS (three subgroups) or 1-MHz FUS (two subgroups). Six animals were included in each of the 0.4- and 1-MHz subgroups except the 0.41 MI/0.65 CI subgroup (n = 4) (total n = 28, see Supplementary Table S1). All contralateral sides which received only microbubbles were denoted as the non-FUS (as 0 MI) group.

### Focused ultrasound

Rats were initially anesthetized with 3% isoflurane in 100% O_2_ and continually maintained with 2% isoflurane in 100% O_2_ during FUS-induced BBB opening. The fur overlying the FUS area was removed for FUS penetration.

The animals were placed in a prone position directly under an acrylic water tank with a 4 × 4 cm^2^ window sealed with a thin polyethylene membrane to allow the ultrasound to penetrate through its base (described in detail in Supplementary Fig. S1A). The space between the skull and the window was filled with ultrasound gel. Lipid-shell Sulfur hexafluoride (SF6) ultrasound microbubbles (2–5 μm mean diameter^23^, 0.2 mL/kg; SonoVue^®^, Bracco Diagnostics Inc., Milan, Italy) and heparin (0.03 ml/kg; Agglutex, China Chemical and Pharmaceutical Corporation, Taipei, Taiwan) were administered intravenously after dilution with normal saline solution to a total volume of 0.3 ml. Immediately following microbubble injection, burst-mode FUS was delivered with a burst length of 10 ms, pulse-repetition frequency of 1 Hz and duration of 90 s. The biological effects induced by this microbubble dosage and FUS pressure have been previously demonstrated^23^,^30^,^31^,^40^. To evaluate the spatial distribution of FUS-induced BBB opening, Evans blue dye (3% in saline, 1 mL/kg) was administrated intravenously following FUS and an MRI contrast agent (Gd-DTPA (0.3 mL/kg; Magnevist^®^, Bayer Schering Pharma, Montville, NJ, USA)) was also administrated intravenously to obtain PD/PK parameters from DCE-MRI after FUS-induced BBB opening. After animal sacrifice, animals conducted Evans blue quantification via spectrophotometric analysis^30^,^31^.

### Dynamic contrast-enhanced MRI (DCE-MRI)

In the *in vitro* measurements in our previous study, the correlation between spin-lattice relaxivity (R1 = 1/T1) mapping and Gd-DTPA concentration was determined using a 7-Tesla MR scanner (Bruker Corp., Billerica, MA, USA)^30^. In the experimental animal groups, the FUS-induced BBB opening was monitored using an MR scanner and a 4-channel surface coil (T7399V3; Bruker Corp., Billerica, MA, USA). Each rat was placed in an acrylic holder, positioned in the center of the magnet, and anesthetized with isoflurane gas (1–2%) at 50–70 breaths/min during the entire MRI procedure.

Following FUS-induced BBB opening, the distribution and dynamics of Gd-DTPA leakage were investigated. Animals were immediately relocated to the MR scanning room, and T1-weighted images of DCE-MRI with multiple flip angles were acquired. R1 maps and Gd-DTPA concentrations were calculated by transferring these multiple flip angle group images (gradient-recall-echo sequence, TR/TE = 2.31 ms/0.76 ms, slice thickness = 0.8 mm; slice number = 14; matrix = 132 × 192, flip angle = 5°/10°/15°/20°/25°/30°)^30^,^31^. Upon completion of the 20th acquisition, a diluted bolus of Gd-DTPA was IV administrated through a catheter at an infusion rate of 6 mL/s. A series of T1-weighted images were sequentially acquired over a period of 60 min. Following the series of DCE T1-weighted images, susceptibility-weighted imaging (SWI) sequences which are able to detect hemorrhages^22^ were obtained to identify possible tissue hemorrhaging associated with MI/CI and FUS-induced BBB opening using the following parameters: TR/TE = 30 ms/18 ms; flip angle = 40°; slice thickness = 0.6 mm; matrix size = 256 × 384; and FOV = 80 × 130 mm^2^.

### DCE-MRI for PD Analysis on FUS-induced BBB opening

The SIs of T1-weighted images from the CE-MRI were obtained at 10 min following Gd-DTPA IV administration (see the time line in Supplementary Fig. S1B). The SI change was computed from the SI of BBB-opening before and after Gd-DTPA administration. The SI change is given by the following equation:


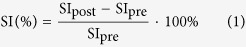


where SI_post_ represents the SI following Gd-DTPA administration, and SI_pre_ represents the SI before Gd-DTPA administration.

The Gd-AUC maps were obtained to characterize the FUS-induced BBB opening. The R1 relaxivity and Gd-DTPA concentration were calibrated *in vitro,* and the linear manner was well presented in our previous study^30^,^31^. Gd-AUC maps were then obtained by accumulating a series of time-dependent Gd-DTPA concentration maps (transferred from R1 maps) to evaluate the PD characteristics of BBB-opening following Gd-DTPA administration (up to 60 min). Thus the total Gd-AUC is given by the following equation:


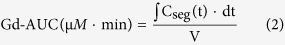


where C_seg_(t) are vertical segments under the Gd-DTPA concentration time curve area and V is total ROI volume.

### DCE-MRI for PK Analysis on FUS-induced BBB opening

Following Gd-AUC observations, other DCE-MRI PK parameters including K_trans_ and V_e_ were obtained to characterize the PK behavior of the FUS-induced BBB opening by analyzing series Gd-DTPA enhanced T1-weighted images within 10 min. The entire BBB-opening/closing process was also investigated by repeating the DCE-MRI image sequence at four time points (10 min, 2 hr, 6 hr, and 24 hr) after FUS-induced BBB opening (time line see Supplementary Fig. S1B). Gd-DTPA concentrations were calculated from SI changes of the T1-weighted image, using conversion equations similar to those used in previous studies^31^. To calculate the kinetic parameters, the Gd-DTPA concentration curve was fit to the extend Kety model^41^,^42^,^43^ which takes into account the presence of separate extracellular and intravascular compartments. The time-dependent concentration of the contrast agent in a tissue can then be described as


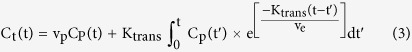


where C_p_(t) is the contrast agent concentration in the blood plasma (i.e. the arterial input function (AIF)), C_t_(t) is the contrast concentration in the tissue, K_trans_ is the transfer rate constant from the intravascular system to the EES, and V_p_ and V_e_ are the capillary plasma volume and distribution volume of contrast agent in the EES (per unit volume of tissue).

The SIs of all rat brains were converted to C_t_(t) values on the Gd-DTPA concentration time curve, and C_p_(t) was chosen from a region of interest (ROI) in the vein sinus. K_trans_ and V_e_ value were fitted pixel-by-pixel, using the least squares function in the Matlab optimization toolbox (MathWorks, Inc., Natick, MA, USA) to generate two different PK parameter maps. For dynamic kinetic evaluation, a circular ROI was assigned at the targeted BBB-opening region to calculate K_trans_/V_e_ values at different time points (10 min, 2 hr, 6 hr, 24 hr). Finally, the exponential time decay curves of K_trans_ and V_e_ were obtained by curve fitting, which was also described in a previously^31^.

### Histology

Previous studies have demonstrated that regions of the FUS-induced BBB opening can be clearly shown by staining with Evans Blue dye^30^,^31^,^44^,^45^. Therefore after the 4th DCE-MRI image sequence (24 hr), all rats were first deeply anesthetized with 35% chloral hydrate and infused with heparinized saline through the cardiac ventricle until a colorless infusion fluid was obtained from the atrium. In each rat, the post-mortem brain was photographed using a digital camera against a standard scale. Brain samples were serially sectioned (2-μm thickness) and stained with hematoxylin and eosin (H&E). Histologic evaluation was performed blind to the ultrasound parameters. However, the observer was informed of the specific sonication side.

### Passive cavitation detection

Since the inertial cavitation and stable cavitation can be characterized by acoustic emissions, to identify their association with BBB-opening and association with MI and CI, passive cavitation detection (PCD) analysis was conducted in an *in vitro* setup to assess the degree of stable cavitation (quantified by stable cavitation dose, SCD) and inertial cavitation (quantified by inertial cavitation dose, ICD) at various acoustic pressures. The 0.4 MHz and 1 MHz transducers were driven by an amplifier (150A100B; AR, Souderton, PA, USA) and a waveform generator (model AWG 2040, Tektronix, CA, USA) to transmit burst-FUS to an agar-based vessel phantom (2% agarose) with a diameter of 1 mm. The duty was 100 cycles and the acoustic pressure was compatible with the transcranial pressure (from 0.26 to 0.88 MPa were for 0.4-MHz FUS and from 0.22 to 0.77 MPa were for 1-MHz FUS). For PCD, a customized focused hydrophone with a bandwidth of 0.01–10 MHz (model Y-134, Sonic Concepts Inc., WA, USA) was placed at a 90° angle to obtain acoustic emissions induced by the stable and inertial cavitation. The obtained signals were digitized using an oscilloscope (model LT354, LeCroyCorp., NY, USA) after being amplified by a broadband receiver (BR-640A, Ritec, Warwick, RI). The signals were converted into spectra with frequency domains using MATLAB software (Mathworks, Natick, MA) to assess SCD and ICD. For each case, the SCD was calculated with the integration of the subharmonic/ultraharmonic frequency band for 0.4-MHz FUS (0.2-, 0.6-, 1-MHz) or 1-MHz FUS (0.5-, 1.5-, 2.5-MHz). The ICD was computed with the integration of multiple frequency bands that fell within the bandwidth of the focused hydrophone but outside the incident and harmonic frequencies of FUS. For both SCD and ICD, the calculated results were normalized to the results of pure water obtained under the same experimental conditions. The experimental setup and quantification method are taken from previous studies^16^,^46^.

### Statistical analysis

Statistical analysis was performed using SPSS software (IBM SPSS statistics; IBM Corp., Armonk, NY, USA) by two researchers blind to animal assignment. DCE-MRI parameter data and PCD data are presented as mean ± standard error of the mean and analyzed by one-way ANOVA. The *p*-value for statistical significance was 0.05. The Fisher’s r to z transformation was performed to estimate the confidence intervals of the difference between correlations gauged by MI and CI.

## Additional Information

**How to cite this article**: Chu, P.-C. *et al*. Focused Ultrasound-Induced Blood-Brain Barrier Opening: Association with Mechanical Index and Cavitation Index Analyzed by Dynamic Contrast-Enhanced Magnetic-Resonance Imaging. *Sci. Rep.* **6**, 33264; doi: 10.1038/srep33264 (2016).

## Supplementary Material

Supplementary Information
