# Supplementary material for: Focused Ultrasound-Induced Blood-Brain Barrier Opening: Association with Mechanical Index and Cavitation Index Analyzed by Dynamic Contrast-Enhanced Magnetic-Resonance Imaging
Source: Sci Rep. 2016 Sep 15;6:33264. doi: 10.1038/srep33264 (PMC5024096; doi:10.1038/srep33264)
Supplement: Supplementary Information [file srep33264-s1.doc]

**Focused Ultrasound-Induced Blood-Brain Barrier Opening: Association with Mechanical Index and Cavitation Index Analyzed by Dynamic Contrast-Enhanced Magnetic-Resonance Imaging**

## Po-Chun Chu^1, +^, Wen-Yen Chai^1, 2, +^, Chih-Hung Tsai^1^, Shih-Tsung Kang^3^, Chih-Kuang Yeh^3^ and Hao-Li Liu^1,4,5^

^1^Department of Electrical Engineering, Chang-Gung University, Taoyuan, 333 Taiwan, ^2^Department of Diagnostic Radiology and Intervention, Chang-Gung Memorial Hospital, Taoyuan, 333 Taiwan, ^3^Department of Biomedical Engineering and Environmental Sciences, National Tsing Hua University, Hsinchu 300, Taiwan, ^4^Department of Neurosurgery, Chang Gung Memorial Hospital, Taoyuan, 333, Taiwan, ^5^Medical Imaging Research Center, Institute for Radiological Research, Chang Gung University and Chang Gung Memorial Hospital, Taoyuan, Taiwan.

**^+^ These authors contributed equally to this work**


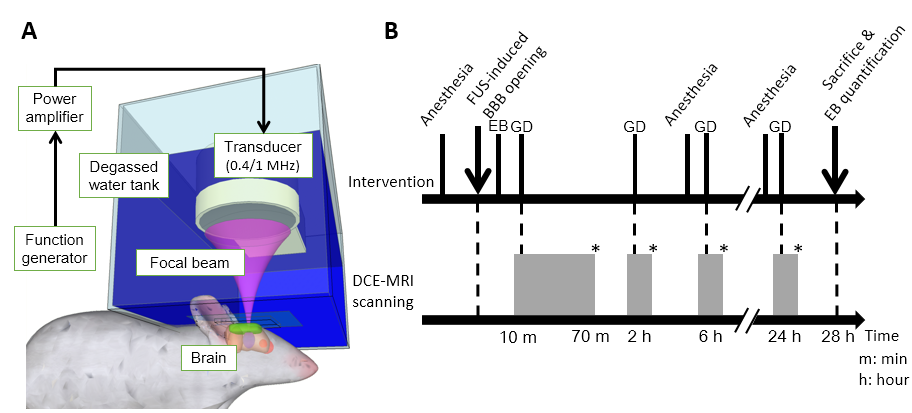


**Figure S1.** Schematic drawing to demonstrate FUS delivery and experimental design. (**A**) FUS delivery set-up. (**B**) Time course of experiments. The T1-weighted images and DCE-MRI image sequence were obtained at four different time points, 10 mins, 2 hrs, 6 hrs, and 24 hrs after FUS-induced BBB opening (Figure 1B).

**Table S1.** Experimental design. Five difference acoustic pressures were used in FUS-induced BBB opening, total 28 animals were separated into each of the 0.4- and 1-MHz subgroups. *f_0_*: exposure frequency; MI: mechanical index; CI: cavitation index; n: animal number in group.

| Group (n) | *f_0_*  (MHz) | Acoustic pressure (with skull decay)  (MPa) | MI | CI |
| --- | --- | --- | --- | --- |
| 1 (4) | 0.4 | 0.26 | 0.41 | 0.65 |
| 2 (6) |  | 0.35 | 0.56 | 0.89 |
| 3 (6) |  | 0.71 | 1.12 | 1.77 |
| 4 (6) | 1 | 0.43 | 0.43 | 0.43 |
| 5 (6) |  | 0.83 | 0.83 | 0.83 |

**Table S2.** Summary of four DCE-MRI parameters to FUS-induced BBB opening. Four DCE-MRI parameters to FUS-induced BBB opening. Figure 1 shows the post-mortem brains stained by Evans blue dye leakage and traditional SI change of the T1-weighted image from DCE-MRI to compare with the DCE-MRI analysis including the Gd-AUC, K_trans_, and V_e_. *f_0_*: exposure frequency; MI: mechanical index; n: animal number in group.

| Group | *f_0_* (MHz) | MI | CI |  | T1 | |  | | Gd-AUC | |  | |
| --- | --- | --- | --- | --- | --- | --- | --- | --- | --- | --- | --- | --- |
|  |  |  |  |  | SI (%) | |  | | Gd-AUC (μM) | |  | |
| 1 | 0.4 | 0.41 | 0.65 |  | | 22.806 ± 5.751 | |  | | 253.962 ± 98.843 | |  |
| 2 | 0.4 | 0.56 | 0.89 |  | | 30.646 ± 11.561 | |  | | 270.776 ± 115.039 | |  |
| 3 | 0.4 | 1.12 | 1.77 |  | | 50.134 ± 12.219 | |  | | 521.063 ± 126.867 | |  |
| 4 | 1 | 0.43 | 0.43 |  | | 30.707 ± 10.886 | |  | | 284.827 ± 61.156 | |  |
| 5 | 1 | 0.83 | 0.83 |  | | 39.714 ± 11.475 | |  | | 474.83 ± 130.71 | |  |

| Group |  | K_trans_ | |  | V_e_ | |
| --- | --- | --- | --- | --- | --- | --- |
|  |  | Mean (min^-1^) | *T*_1/2_ (hrs) |  | Mean | *T*_1/2_ (hrs) |
| 1 |  | 0.0063 ± 0.0002 | 2.67 |  | 0.0285 ± 0.0069 | 1.02 |
| 2 |  | 0.0092 ± 0.0016 | 2.48 |  | 0.0533 ± 0.0083 | 1.68 |
| 3 |  | 0.0136 ± 0.0017 | 4.34 |  | 0.0787 ± 0.0158 | 3.69 |
| 4 |  | 0.0061± 0.0009 | 2.47 |  | 0.0398 ± 0.0092 | 1.65 |
| 5 |  | 0.0095 ± 0.0025 | 3.24 |  | 0.0616 ± 0.0123 | 2.35 |

**Table S3.** Summary of four correlation coefficient between four DCE-MRI parameters and MI or CI. The MI and CI both well correlated to DCE-MRI parameters for either 0.4 or 1 MHz exposure. The Fisher’s r to z transformation showed that the correlation coefficients between MI and DCE-MRI parameters were better than CI and DCE-MRI parameters for both exposure frequencies. However, there is no significant difference for correlations between BBB opening and MI and CI (*p* > 0.05 for four DCE-MRI parameters)

| Index |  | Correlation coefficient (for MI) | | |  | Correlation coefficient (for CI) | | |
| --- | --- | --- | --- | --- | --- | --- | --- | --- |
|  |  | 0.4 MHz | 1 MHz | 0.4/1 MHz |  | 0.4 MHz | 1 MHz | 0.4/1 MHz |
| T1 |  | 0.996 | 0.9371 | 0.9682 |  | 0.9964 | 0.9371 | 0.8481 |
| Gd-AUC |  | 0.9876 | 0.9993 | 0.9666 |  | 0.9869 | 0.9993 | 0.7951 |
| K_trans_ |  | 0.9785 | 0.9989 | 0.9684 |  | 0.9794 | 0.9989 | 0.9396 |
| V_e_ |  | 0.9461 | 0.9898 | 0.9333 |  | 0.9467 | 0.9898 | 0.8291 |

|  |  | Fisher’s r to z transformation | | | | | | | |
| --- | --- | --- | --- | --- | --- | --- | --- | --- | --- |
|  |  | MI to CI for 0.4 MHz | |  | MI to CI for 1 MHz | |  | MI to CI for both 0.4/1 MHz | |
| Index |  | Z | *p* value |  | Z | *p* value |  | Z | *p* value |
| T1 |  | 0 | 1 |  | 0 | 1 |  | 1.15 | 0.2501 |
| Gd-AUC |  | 0 | 1 |  | 0 | 1 |  | 1.35 | 0.177 |
| K_trans_ |  | -0.02 | 0.984 |  | 0 | 1 |  | 0.47 | 0.6384 |
| V_e_ |  | -0.01 | 0.992 |  | 0 | 1 |  | 0.71 | 0.2113 |


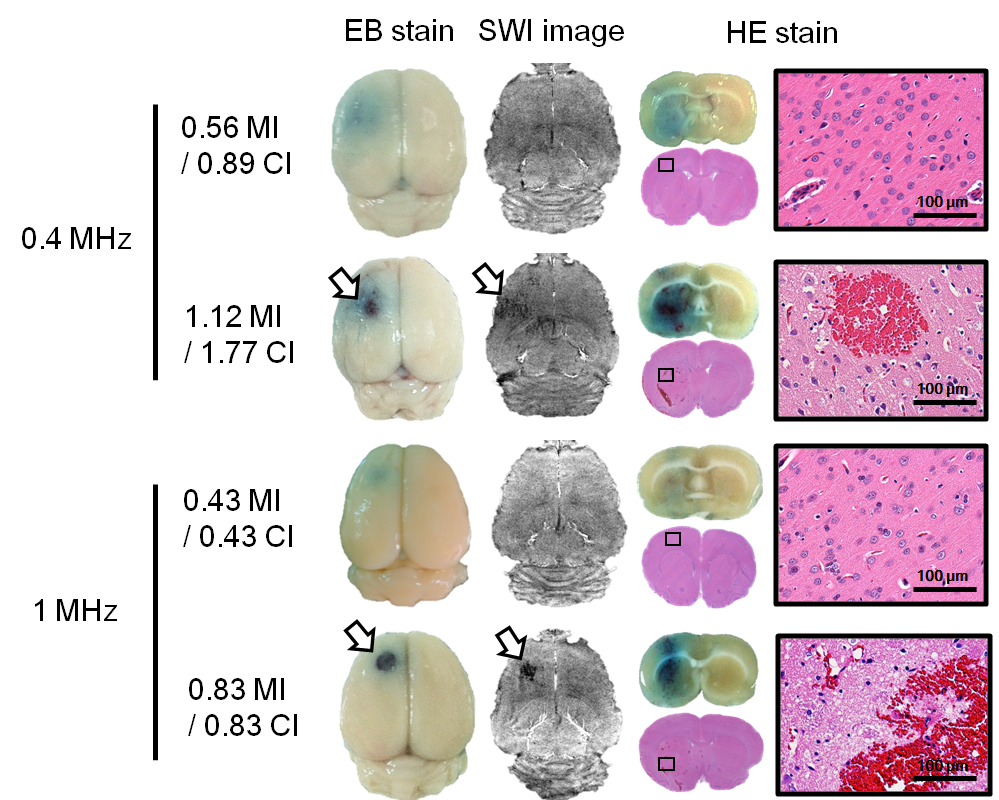


**Figure S2.** Representative gross views of EB-stained brains, SWI image and HE stain at various MI/CI exposure levels. For exposure level increased to exceed 0.6-MI, both the 1- and 0.4MHz FUS exposure induced BBB-opening accompanied with noticeable erythrocytes extravasations.


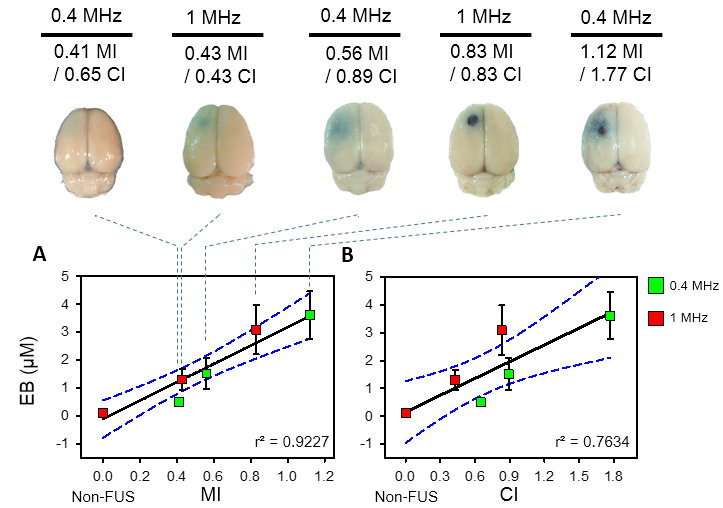


**Figure S3.** Gd-DTPA enhanced EB stain maps and correlations of MI/ CI with EB concentration. EB concentration was increased as a function of MI/CI change. (**A**) The correlation between MIs and EB concentration. The non-FUS side serves as 0 MI. The EB concentration was monotonically increased as a function of MI change regardless of exposure frequency (r^2^ = 0.9227). (**B**) The correlation between CIs and EB concentration. The non-FUS side serves as 0 CI. The correlation of CI and EB concentration decreased but was still sufficiently high (r^2^ = 0.7634).


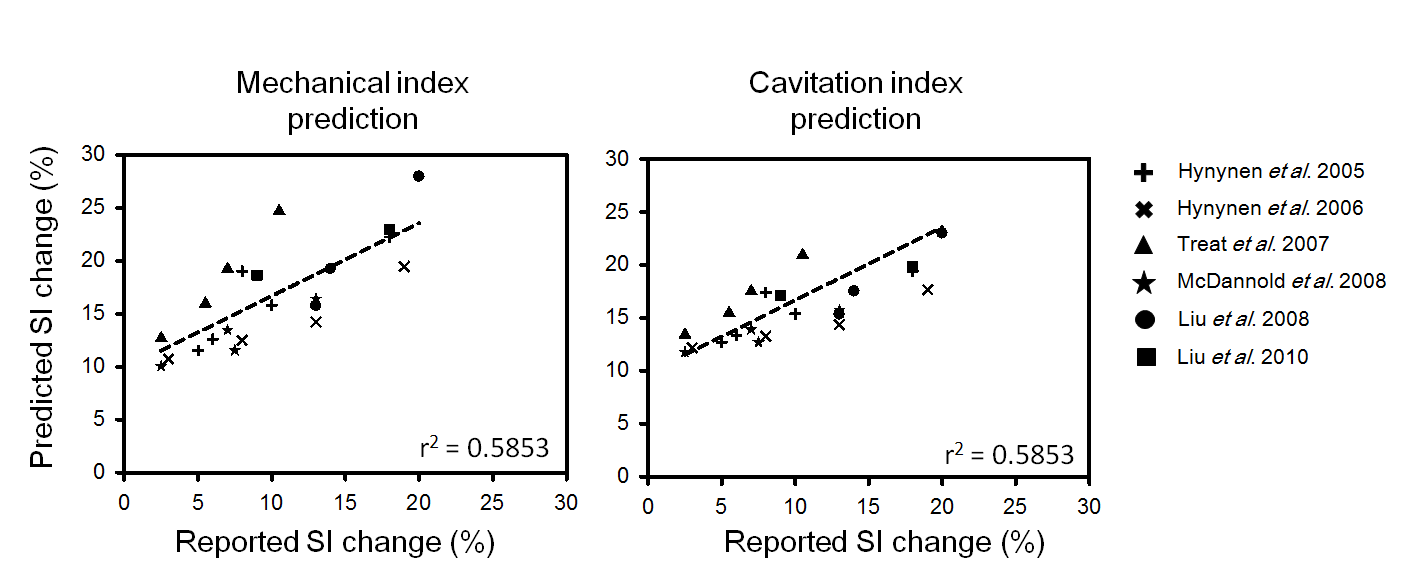


**Figure S4.** The correlation between the predicted SIs and the reported SIs from previous studies. The MI-SI-correlated equation contributed high correlation with reported SIs. ( Hynynen *et al*. 2005 ^1^, Hynynen *et al*. 2006 ^2^, Treat *et al*. 2007 ^3^, McDannold *et al*. 2008 ^4^, Liu *et al*. 2008 ^5^, Liu *et al*. 2010 ^6^)

**REFERENCE**

1 Hynynen, K., McDannold, N., Sheikov, N. A., Jolesz, F. A. & Vykhodtseva, N. Local and reversible blood-brain barrier disruption by noninvasive focused ultrasound at frequencies suitable for trans-skull sonications. *NeuroImage* **24**, 12-20, doi:10.1016/j.neuroimage.2004.06.046 (2005).

2 Hynynen, K. *et al.* Focal disruption of the blood-brain barrier due to 260-kHz ultrasound bursts: a method for molecular imaging and targeted drug delivery. *Journal of neurosurgery* **105**, 445-454, doi:10.3171/jns.2006.105.3.445 (2006).

3 Treat, L. H. *et al.* Targeted delivery of doxorubicin to the rat brain at therapeutic levels using MRI-guided focused ultrasound. *International journal of cancer. Journal international du cancer* **121**, 901-907, doi:10.1002/ijc.22732 (2007).

4 McDannold, N., Vykhodtseva, N. & Hynynen, K. Blood-brain barrier disruption induced by focused ultrasound and circulating preformed microbubbles appears to be characterized by the mechanical index. *Ultrasound in medicine & biology* **34**, 834-840, doi:10.1016/j.ultrasmedbio.2007.10.016 (2008).

5 Liu, H. L. *et al.* Hemorrhage detection during focused-ultrasound induced blood-brain-barrier opening by using susceptibility-weighted magnetic resonance imaging. *Ultrasound in medicine & biology* **34**, 598-606, doi:10.1016/j.ultrasmedbio.2008.01.011 (2008).

6 Liu, H. L. *et al.* Blood-brain barrier disruption with focused ultrasound enhances delivery of chemotherapeutic drugs for glioblastoma treatment. *Radiology* **255**, 415-425, doi:10.1148/radiol.10090699 (2010).
